# Second edition ICCR dataset for testicular germ cell tumours: a reporting guide for histopathological diagnosis of orchiectomy specimens

**DOI:** 10.1111/his.70041

**Published:** 2025-12-12

**Authors:** Felix Bremmer, Fleur Webster, Gedske Daugaard, Robert J. Hamilton, Muhammad T. Idrees, Chia‐Sui Kao, Kosuke Miyai, Maria Rosaria Raspollini, John R. Srigley, Satish Tickoo, Asli Yilmaz, Thomas Wagner, Daniel M. Berney

**Affiliations:** ^1^ Institute of Pathology University Medical Center Göttingen Göttingen Germany; ^2^ International Collaboration on Cancer Reporting Surry Hills NSW Australia; ^3^ Department of Oncology Copenhagen University Hospital, Rigshospitalet Copenhagen Denmark; ^4^ Princess Margaret Cancer Centre Toronto ON Canada; ^5^ Department of Pathology and Laboratory Medicine Indiana University School of Medicine Indianapolis IN USA; ^6^ Diagnostics Institute, Laboratory Medicine—Anatomic Pathology, Cleveland Clinic Cleveland OH USA; ^7^ Department of Pathology and Laboratory Medicine National Defense Medical College Hospital Saitama Japan; ^8^ Department of Histopathology and Molecular Diagnostics Careggi University Hospital Florence Italy; ^9^ Department of Laboratory Medicine and Pathobiology University of Toronto Toronto ON Canada; ^10^ Memorial Sloan Kettering Cancer Center New York NY USA; ^11^ Department of Pathology, Calgary Laboratory Services University of Calgary Calgary AB Canada; ^12^ Department of Pathology Copenhagen University Hospital, Herlev and Gentofte Hospital Herlev Denmark; ^13^ Centre of Cancer Biomarkers and Biotherapeutics, Barts Cancer Institute Queen Mary University of London London UK

**Keywords:** classification, dataset, germ cell tumours, ICCR, prognostic factors, testis

## Abstract

To summarise the content and significance of the recently published second edition International Collaboration on Cancer Reporting (ICCR) histopathology dataset for testicular germ cell tumours, covering the Orchiectomy specimen dataset. We highlight key updates from the first editions, including alignment with the 5th edition World Health Organization (WHO) Classification, revised staging criteria, clarified core data elements versus non‐core elements and the evidentiary basis underpinning these changes. A review of the ICCR 2nd edition dataset for Orchiectomy specimens of primary testicular tumours was performed, focusing on their development by an international expert committee using a consensus‐based approach. Core (required) and non‐core (recommended) data elements were identified along with the level of evidence supporting each, following National Health and Medical Research Council (NHMRC) criteria. Changes from the first edition were extracted by comparing dataset content and notes, informed by up‐to‐date literature through July 2024. The 2nd edition Orchiectomy dataset provides an integrated, harmonised framework for reporting testicular germ cell tumours. The dataset incorporates the WHO 5th Edition Classification of Urinary and Male Genital Tumours. Pathological staging criteria have been updated to align with the 8th edition Union for International Cancer Control (UICC)/American Joint Committee on Cancer (AJCC) tumour‐node‐metastasis (TNM) definitions. The second edition of this dataset includes changes to align the dataset with the WHO Classification of Tumours, Urinary and Male Genital Tumours, 5th edition, 2022. The ICCR dataset includes the 5th edition Corrigenda, July 2024. It was agreed that this dataset is not suitable for non‐germ cell tumours, with the hope that a new dataset, especially for sex‐cord stromal tumours, would be developed. The 2nd edition Orchiectomy dataset represents an authoritative, up‐to‐date standard for pathology reporting of primary testicular germ cell tumours. By incorporating the WHO 5th edition classifications, current TNM staging and the latest evidence on prognostic factors, this dataset facilitates uniform reporting and prognostication. The ICCR dataset underscores core data required for patient management decisions (e.g., adjuvant therapy in Stage I disease, post‐chemotherapy management) while providing flexibility through non‐core elements for additional useful information. Adoption of this internationally vetted dataset will enhance consistency, assist multidisciplinary treatment planning and align pathology reports with modern consensus guidelines and classifications. The dataset can be used in both high‐resource and limited‐resource settings without compromising the essential reporting standards.

## Graphical abstract

The current 2nd edition Orchiectomy dataset offers a rigorously validated, internationally developed checklist that supports high‐quality, standardised reporting and promotes clinical confidence across diverse healthcare settings. All ICCR datasets are freely available to the global pathology community via the ICCR website ([www.iccr‐cancer.org](http://www.iccr‐cancer.org)), reinforcing their role as a key international resource.

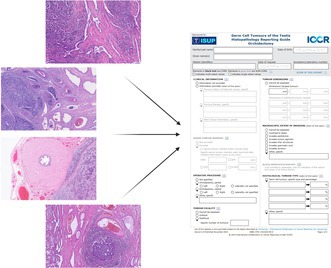

**Abbreviations**
AJCC: American Joint Committee on Cancer
DAC: Dataset Authoring Committee
GCNIS: germ cell neoplasia in situ
GCTs: testicular germ cell tumours
ICCR: International Collaboration on Cancer Reporting
ISUP: International Society of Urological Pathology
LVI: lymphovascular invasion
NHMRC: National Health and Medical Research Council
TNM: tumour‐node‐metastasis
UICC: Union for International Cancer Control
WHO: World Health Organization

## Introduction

Testicular germ cell tumours (GCTs) are a paradigm of a curable malignancy, but their management requires precise pathological evaluation of both the primary tumour and any metastatic disease. Standardised pathology reporting is essential to ensure that all prognostically critical information is captured and communicated to clinicians. Historically, significant variability existed in how pathologists handled and reported testicular tumours, as shown by international surveys of practice.^1^ In response, the International Collaboration on Cancer Reporting (ICCR) developed evidence‐based, a consensus dataset to guide the uniform reporting of testicular cancer pathology. The first edition of the ICCR orchiectomy (radical/partial orchiectomy) dataset was introduced to establish a minimum reporting standard and reduce inter‐observer variation. Since those initial publication, there have been important developments that prompted dataset revision—notably, the release of the WHO 5th Edition Classification of Urinary and Male Genital Tumours (2022) with updated taxonomy and criteria,^2^, ^3^ as well as new clinical studies clarifying prognostic factors in stage I.^4^, ^5^ In late 2024, the ICCR published second edition reporting guides for testicular cancer, comprising separate but complementary dataset for orchiectomy specimens.^2^, ^6^ This updated dataset was developed by an international committee of expert urological pathologists and oncologists to reflect the latest consensus in the field and provides a comprehensive overview of the second edition ICCR testicular tumour dataset. We outline their scope and content, emphasise changes from the first editions and discuss the rationale and evidence underlying these changes. The dataset present in an integrated narrative to highlight how together they form a continuum of information critical for staging and managing testicular GCT patients. Key themes include the delineation of core (required) versions non‐core (recommended) data elements based on level of evidence and consensus. This includes alignment with current WHO Classification and tumour‐node‐metastasis (TNM) staging, incorporation of prognostic factors (with distinction between those well‐founded in evidence and those still under evaluation) and the ultimate clinical relevance of the reported pathological features in guiding patient therapy and follow‐up.

## Methods

### Dataset Development and Evidence Review

The ICCR dataset update process followed a structured approach combining evidence‐based medicine with expert consensus, a two‐month global consultation and oversight by the ICCR Dataset Steering Committee. The Dataset Authoring Committee (DAC) for testis tumours comprised international specialists in genitourinary pathology and oncology, who reviewed the literature up to July 2024 and considered emerging data and collective clinical experience. Data elements were classified as core if they are essential for patient management, staging or prognosis. This designation requires a foundation of at least level III‐2 evidence (observational studies with low risk of bias) according to the National Health and Medical Research Council (NHMRC) prognostic evidence hierarchy,^7^ or, in the absence of high‐level evidence, unanimous agreement by the DAC that the element is critical for inclusion. Elements that do not meet the criteria for core inclusion but were felt to provide useful or important additional information, were designated as non‐core (optional)—these may reflect good practice or emerging factors not yet validated to the same degree. The summation of all core elements in a report is intended to represent the minimum essential dataset for testicular cancer pathology. For instance, pathological features with proven impact on outcomes (supported by robust studies or meta‐analyses) are core, whereas features of more investigational or minor importance are non‐core (even though they may still be reported for completeness or research interest). Importantly, the ICCR encourages collection of non‐core data, as today's non‐core element may gain evidence in the future to become core. The dataset authors reviewed the text of the ICCR 2nd edition Orchiectomy dataset, including explanatory notes and referenced literature. We identified points of change or clarification relative to the prior edition by expert comparison of content (the first edition of this dataset, published in 2017, was used as a baseline). Particular attention was paid to updates driven by new evidence (for example, recent large cohort studies on stage I prognosticators) and to modifications aligning with external standards (WHO classification changes, updates in the TNM staging system and other international reporting protocols). No separate patient data were involved in this analysis; it is a synthesis of published guidelines and literature.

## Results and Synthesis

### Overview of the Second Edition Orchiectomy Dataset

The ICCR second edition Orchiectomy dataset (for radical or partial orchiectomy specimens of the testis) serves complementary roles. The orchiectomy dataset addresses the primary testicular tumour, encompassing germ cell tumours in the testis (in patients of any age) and their in situ precursors. Paratesticular malignancies and sex cord‐stromal tumours of the testis are outside the scope of these dataset (the latter have distinct criteria for malignancy and are not included, given their complexity and benign vs. malignant behaviour spectrum).^6^, ^8^ A comparative summary of major updates in the second edition vs. first edition for the ICCR Orchiectomy dataset is provided in Table 1.

**Table 1:** Summary of key changes of the ICCR second edition dataset for testicular germ cell tumours (Orchiectomy): The table summarises key updates in classification, staging, core vs. non‐core elements and terminology. ‘2nd ed.’ refers to the November 2024 ICCR dataset

Aspect	Orchiectomy dataset—2nd ed.
WHO Classification	Inclusion of WHO 2022 Corrigenda changes for testis tumours.^2^, ^3^ Updated terminology (e.g., *spermatocytic tumour*, *ENT*) Explicit separation of GCNIS‐derived vs. non‐GCNIS tumour categories
Pathologic staging (TNM)	Fully updated to TNM 8th ed. (UICC/AJCC 2017).^9^, ^10^ Epididymis and hilar soft tissue invasion is pT2. AJCC8 seminoma pT1a/pT1b (size‐based) acknowledged (not in UICC). Rete testis invasion is not a TNM factor for GCT, but reported for risk stratification. ‘y’ prefix used for post‐chemotherapy orchiectomy cases.
Core data (required) elements	These refinements ensure all prognostic features (even if not in TNM) are captured. Tumour size is measured for all (partly influences staging for seminoma). Rete testis invasion in seminoma explicitly reported (distinction of stromal vs. pagetoid). LVI reaffirmed as core (especially predictive in NSGCT^4^). GCNIS presence set as core (absence prompts consideration of alternate tumour origin). Spermatic cord invasion definition refined. Scrotal invasion noted if present. Margin status core (partial or radical).
Non‐core (optional) elements	Non‐core items serve to enrich the report without being essential. e.g., Tunica albuginea invasion is recorded (no staging impact). Tumour focality (e.g., bilateral tumours) noted if applicable. Marker levels (pre‐orchiectomy serum AFP, HCG, LDH) can be included for context (since they define S category),^11^, ^12^ but are non‐core as lab results. Coexistent pathology (atrophy, GCNIS in contralateral biopsy, etc.) and ancillary study results (IHC, ploidy/FISH).
Prognostic factor updates	*New high‐level evidence*: studies clarifying risk factors in Stage I seminoma vs. NSGCT.^4^, ^5^ *Seminoma*: size ≥3–4 cm and rete invasion are acknowledged as risk factors but with caveats (recent data suggest that pre‐orchiectomy serum markers LDH, β‐hCG may outperform them^5^). *NSGCT*: LVI confirmed as the dominant predictor of relapse.^4^ Presence (and predominance) of embryonal carcinoma also critical as well as soft tissue invasion and size of tumour.^4^ These findings are reflected in notes, potentially shifting some elements from non‐core to core.
Terminology and other	GCNIS (germ cell neoplasia in situ) is the uniform term. Clarifies that ‘pagetoid spread to rete’ is not termed ‘invasion’. Encourages describing uncommon scenarios clearly (e.g., ‘burnt‐out tumour’ for regressed GCT, presence of gonadoblastoma in dysgenetic testis, etc.).

AFP, Alpha‐Fetoprotein; AJCC, American Joint Committee on Cancer; ENT, embryonic‐type neuroectodermal tumour; FISH, Fluorescence In Situ Hybridisation; GCNIS, germ cell neoplasia in situ; HCG, Human Chorionic Gonadotropin; IHC, Immunohistochemistry; LDH, Lactate Dehydrogenase; LVI, lymphovascular invasion; NSGCT, Nonseminomatous Germ Cell Tumour; UICC, Union for International Cancer Control; WHO, World Health Organization.

#### Pathological staging updates

The dataset has been brought in line with the 8th edition TNM staging system (UICC/AJCC 2016–2017),^9^, ^10^, ^11^, ^12^ which had some changes from prior editions. In the Orchiectomy dataset, the definitions of pT categories have been updated. Notably, invasion into the epididymis or into the hilar soft tissues of the testis is now assigned pT2, reflecting a change from earlier staging manuals where epididymal involvement was considered as organ‐confined (pT1) disease. This change arose from evidence that tumour extension into paratesticular soft tissue (which often includes the epididymal connective tissue at the hilar region) portends a higher risk of metastatic disease, and it aligns with recommendations from the International Society of Urological Pathology (ISUP) consultation conference and adoption in AJCC 8th edition.^13^, ^14^ Consequently, the second edition dataset no longer stages isolated epididymal invasion as pT1, as might have been the case in older reports, but as pT2 to reflect current TNM. Likewise, invasion of the tunica vaginalis (the mesothelial sac around the testis) remains pT2—the dataset retains the definition that only true invasion through the single layer of visceral tunica vaginalis constitutes pT2, and not mere adherence or artefactual involvement.

For seminomatous tumours, AJCC 8th edition introduced a subdivision of pT1 based on tumour size (pT1a for tumour <3 cm, pT1b for ≥3 cm) in recognition of size as a prognostic factor in stage I seminoma.^5^ The ICCR dataset acknowledges this by prompting measurement of tumour size and allowing documentation of pT1a/pT1b when reporting in an AJCC context. However, it is noted that this size subdivision was not adopted by UICC TNM and does not change overall stage grouping in the current UICC system.^11^ The dataset therefore includes tumour maximum dimension as an important data point (recorded for all tumours), but cautions that its prognostic weight, particularly for seminoma, may be less than previously thought once other factors are considered.^5^ Neither rete testis invasion nor tumour size was part of TNM 7th edition staging^15^, ^16^ but were often used clinically to guide adjuvant therapy decisions in Stage I seminoma. Their inclusion in AJCC 8th edition reflects earlier studies suggesting increased relapse risk with larger tumours and stromal rete invasion.^10^ The ICCR 2nd edition retains reporting of rete involvement and size so that this information is available for clinical risk stratification if needed, and in recognition of European guidelines that still incorporate these factors.^17^, ^18^ At the same time, the accompanying notes update the evidence: recent high‐volume studies have shown that stromal invasion of the rete testis (tumour breaching into the rete stroma) is an independent risk factor for relapse, whereas mere pagetoid (‘in situ’) involvement of the rete epithelium is not prognostically significant. Pathologists are now advised to distinguish these two patterns—only the former constitutes true invasion. In fact, Wagner *et al*. (2024) found that pagetoid rete involvement in seminomas did not correlate with recurrence, whereas genuine rete stromal invasion did.^5^ Rete testis stromal invasion is still considered as pT1 even though there is evidence that stromal invasion is an independent risk factor for relapse. This clarification is new in the second edition and was less clear in the first edition. The dataset authors have accordingly emphasised that pagetoid spread into rete or stromal rete invasion is pT1‐category and only stromal rete invasion is an independent risk factor for recurrence.^5^

### Key Core Data Elements and Changes in the Orchiectomy Dataset

**Figure 1:**
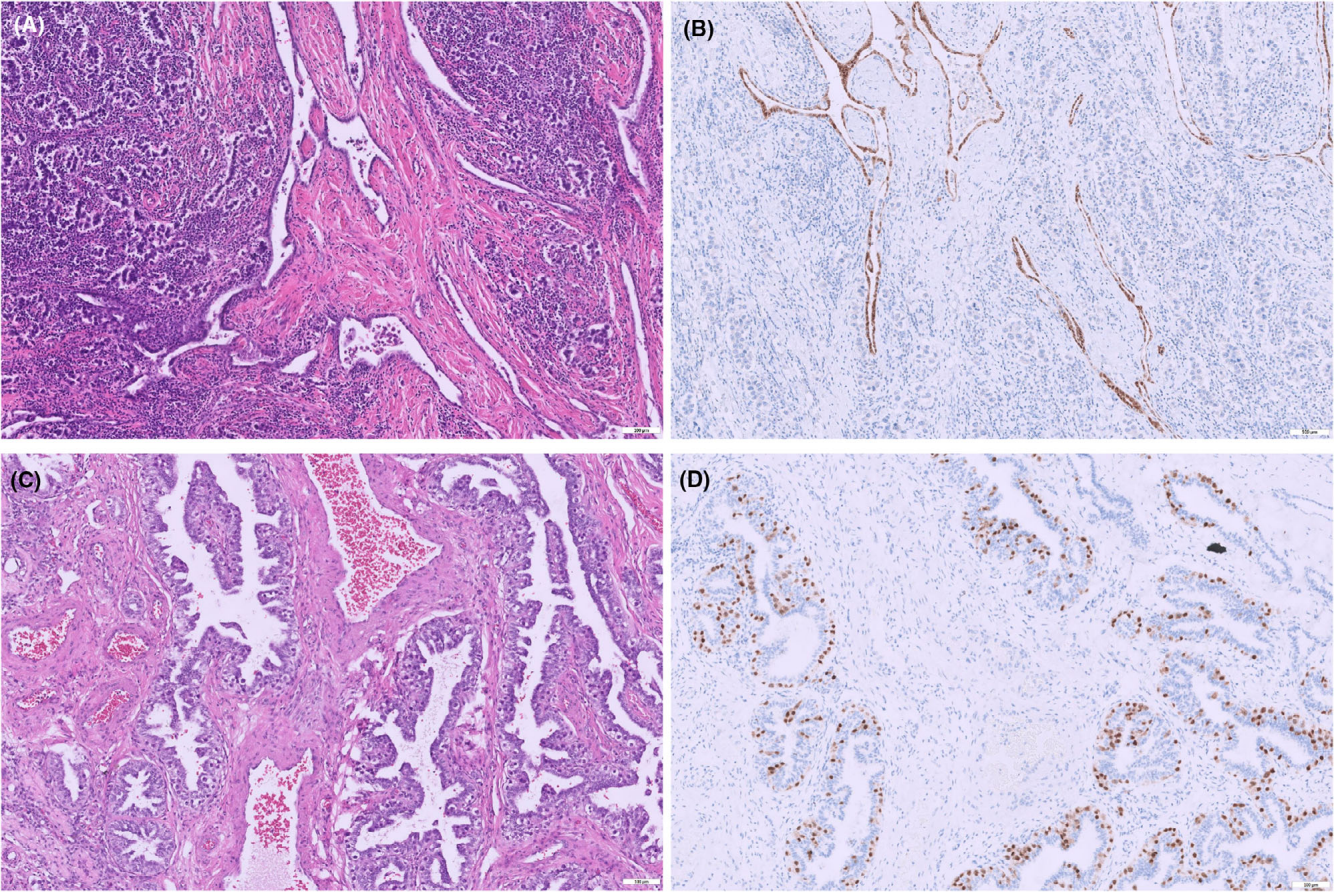
Rete testis invasion: The seminoma infiltrates the stroma of the rete testis (**A**, HE); the epithelium of the rete testis is positive for PAX‐8 (**B**). Non‐invasive germ cells can show a pagetoid involvement of the rete testis without stroma invasion (**C**, HE; **D**, OCT3/4).

**Figure 2:**
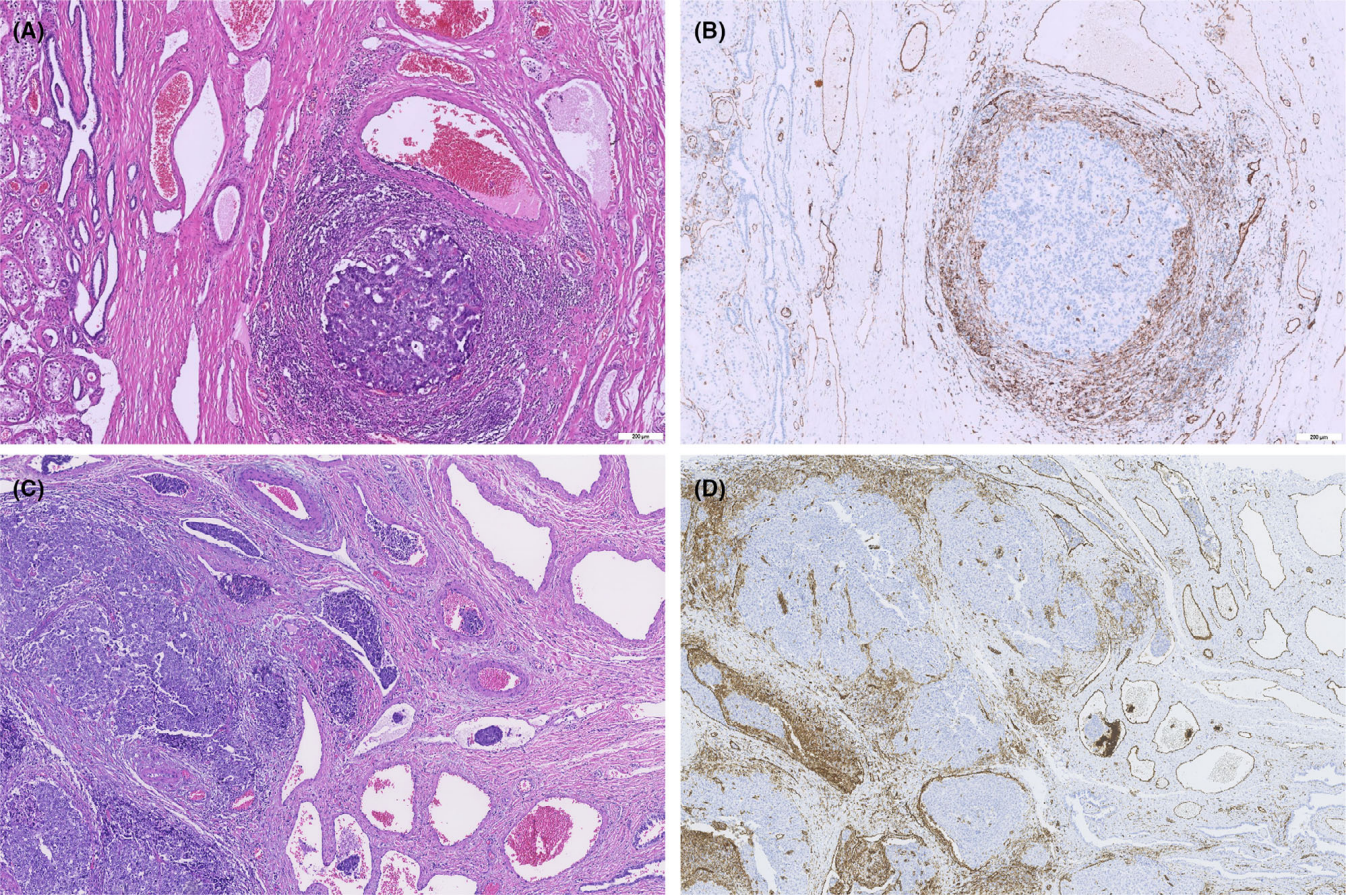
Vascular invasion of germ cell tumours: Vascular invasion is particularly important for staging. It can be challenging in individual cases and requires immunohistochemical analysis. Morphologically, vascular invasion is suspected (**A**, HE), but CD31 staining does not detect any vascular infiltration (**B**; CD31). In contrast, vascular invasion can be clearly recognisable morphologically, which is confirmed by immunohistochemistry (**C**, HE; **D**, CD31).

**Figure 3:**
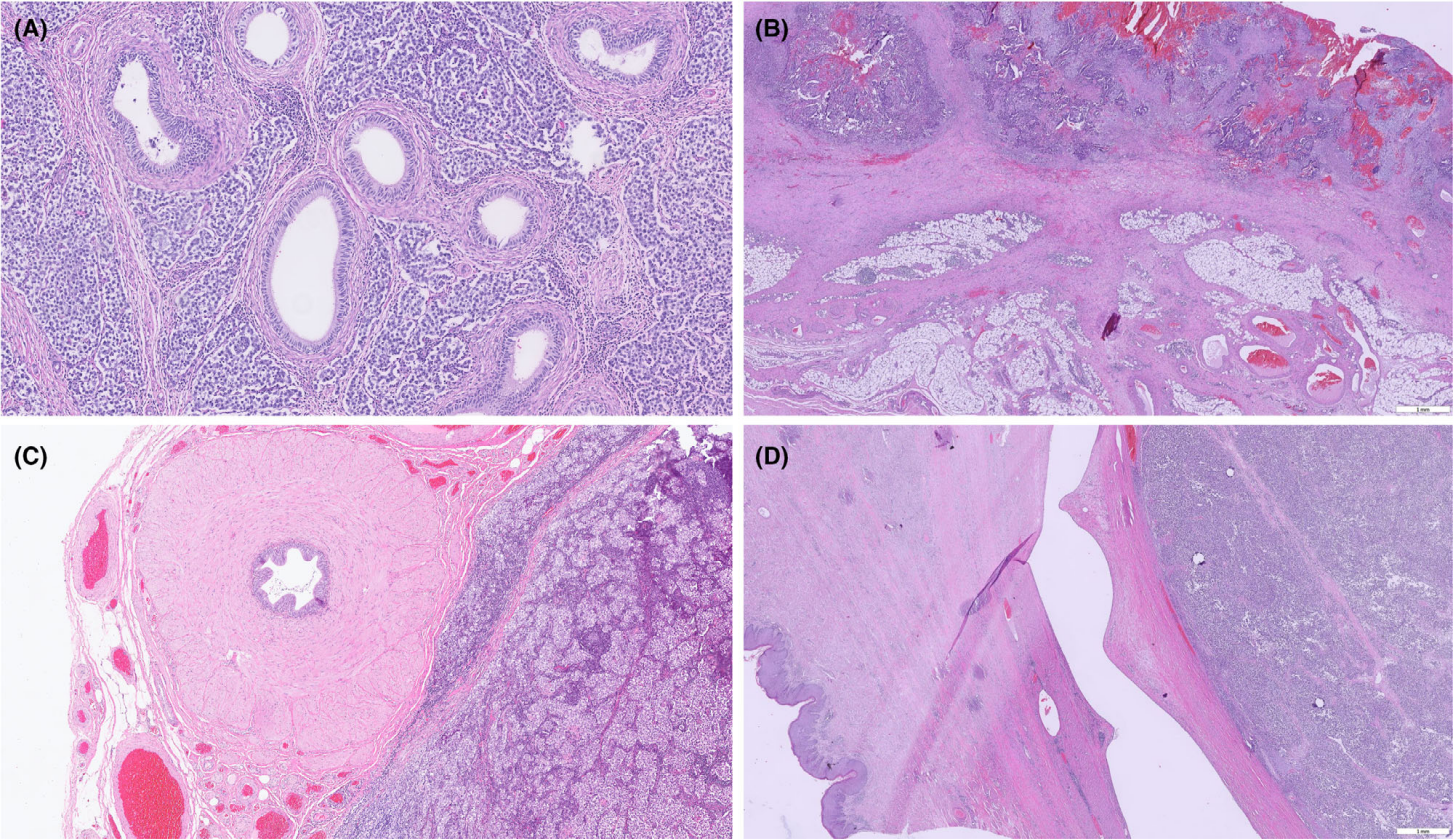
Histopathological findings that influence the T‐category: Seminoma infiltrates the epididymis (**A**, HE), and non‐seminoma with infiltration of the tunica vaginalis (**B**, HE) indicates a pT2‐category. Seminoma with infiltration of the spermatic cord (**C**; HE) indicates pT3‐category. Seminoma with incipient infiltration of the scrotal skin (**D**; HE) indicates pT4‐category.

In the primary tumour (orchiectomy) dataset, the core elements cover all information needed to accurately classify the tumour and assess its extent within the testis and surrounding paratesticular structures. Many of these remain unchanged from the first edition, but several have been clarified or given added emphasis in light of new evidence:

- *Tumour type* (*histological diagnosis*): Every tumour must be typed according to the 2022 WHO classification. Mixed GCTs should have their components identified and ideally quantified by percentage. The second edition aligns with WHO 5th edition, meaning pathologists will use updated terminology (e.g., ‘post‐pubertal teratoma’ vs ‘prepubertal‐type teratoma’) and recognise unusual malignant transformations in a GCT (such as somatic malignancies) by their specific type. This element was, and remains, core. The dataset emphasises documenting certain histopathological features because of clinical impact—for instance, noting the presence of an embryonal carcinoma component in a mixed GCT is important, as embryonal carcinoma is aggressive and its presence (especially with lymphovascular invasion (LVI)) strongly predicts occult metastases.^4^, ^19^ A meta‐analysis confirmed that in clinical stage I non‐seminomatous GCT (NSGCT), the presence of embryonal carcinoma and LVI confers a significantly higher risk of micrometastatic disease.^19^ More recently, Wagner *et al*. (2024) reaffirmed the prognostic importance of embryonal carcinoma. They demonstrated that incorporating embryonal carcinoma in a simple‐to‐use three‐tier categorisation—absent, non‐predominant or predominant (with predominant defined as the histologic subtype present in the greatest proportion) markedly improved risk stratification compared to the binary approach (absent vs. present).^4^ Therefore, the report should clearly indicate if these high‐risk components are present and estimate the proportion of embryonal carcinoma when present.
- *Tumour size*: The maximum dimension of the tumour is recorded (in millimetres). The maximum diameter of the largest tumour is a core measurement. The dataset authors recommend that when there is multifocality, the largest diameter of the largest focus be recorded, and that the maximum diameter of the additional nodules may also be recorded (non‐core). This was recommended in the first edition and is now explicitly reinforced because of its incorporation in seminoma staging (AJCC pT1a/b) and its historical role as a risk factor. In stage I seminoma, tumour size ≥4 cm was long associated with higher relapse rates (approximately doubling the risk) based on earlier studies.^11^, ^20^ The pooled analysis by Warde *et al*. (2002) is often cited for establishing tumour size >4 cm and rete testis invasion as risk factors for relapse in surveillance patients.^10^ However, contemporary analyses have nuanced this view: for example, a 2024 Danish nationwide study found that when factoring in modern pathology review and pre‐orchiectomy serum markers, tumour size on its own was not an independent predictor of recurrence.^5^ Despite this, tumour size remains an informative data point and is required for AJCC staging classification and has recently been shown to have prognostic significance in NSGCT^4^; therefore, it continues to be captured as a core element. The dataset notes caution that size should be interpreted in context—e.g., very large seminomas might correlate with higher stage simply because larger tumours are more likely to have invaded the rete testis or vessels.
- *Rete testis invasion*: The presence or absence of tumour invasion into the rete testis is recorded. In the new dataset, a crucial refinement is made: only stromal (substantive) rete invasion counts as true invasion (Figure 1A,B). In contrast, pagetoid spread of in situ germ cells into the rete epithelium is biologically akin to GCNIS and should be reported as such (often as GCNIS involvement of rete) rather than as invasive disease (Figure 1C,D). The change reflects evidence that pagetoid rete involvement does not carry the adverse prognosis that genuine rete invasion does.^5^ Rete testis invasion has been controversial as a prognosticator: some earlier studies did not find it independently predictive when other factors were controlled for,^9^, ^21^ but others (with thorough pathology review) did find stromal rete invasion to correlate with relapse risk in seminoma.^5^ The ICCR authoring committee unanimously agreed it remains important to report rete testis invasion. In fact, both rete testis invasion and tumour size, albeit imperfect predictors on their own, are used in many centres to decide on adjuvant therapy vs. surveillance in stage I seminoma and are mentioned in European guidelines.^17^, ^18^ Therefore, the dataset continues to list rete testis invasion as a core item (it does not affect TNM T category in UICC 8th edition, but it is a clinically relevant parameter). The new edition's guidance likely elevates the clarity of reporting (pagetoid vs stromal) rather than introducing the element de novo. Stromal invasion of the rete testis is still pT1 and should not be upstaged to pT2.

- *Lymphovascular invasion* (*LVI*): Detection of LVI within the testicular parenchyma or paratesticular tissue including the spermatic cord is a CORE element, as in the first edition, given its strong association with metastatic spread. The second edition reinforces this by citing multiple studies and noting that LVI is ‘an extremely strong predictor’ of occult metastatic disease. This holds particularly true in non‐seminomatous tumours—the presence of LVI in the primary NSGCT is the single most powerful pathologic predictor of relapse, identified in numerous series and confirmed by the recent Danish cohort study.^4^ Even in seminomas, modern studies that carefully exclude artefacts have demonstrated LVI to be significantly associated with higher stage at presentation and relapse risk.^5^, ^8^, ^22^ Therefore, the dataset advises a diligent search for LVI on haematoxylin and eosin (H&E) sections (with optional use of immunohistochemistry such as endothelial markers in challenging cases Figure 2) and unequivocal reporting (simply ‘present’ or ‘not identified’—avoiding equivocal language).^23^ The second edition did not change the status of LVI (it was a core element in the first edition), but new evidence has further solidified its importance in seminoma as well, and this is reflected in the commentary. It is recommended which *histologic component* is invading vessels in mixed GCT as they may confer different recurrence risks.

- *Extent of tumour invasion* (pathologic T‐category features): The dataset enumerates the structures that may be invaded by the tumour, each of which can affect the pT category. This includes: tunica vaginalis, hilar soft tissue, epididymis, spermatic cord and scrotal wall. In the second edition, the significance of each is discussed. Tunica albuginea invasion (without vaginalis involvement) has no impact on staging (still pT1) and has not been shown to independently predict metastasis on multivariate analysis.^4^, ^5^ Tunica vaginalis invasion (pT2) is rare but upstages the tumour; hence, it must be reported. Epididymal and hilar soft tissue invasion are now clearly recognised as pT2; hence, pathologists are prompted to examine the hilar region carefully. The presence of a tumour in the epididymis should be commented on, and in practice, this finding usually implies the tumour extended through the rete testis into the hilar soft tissue where the epididymis is located. The dataset's clarification here is essentially an update aligning with UICC/AJCC 8th editions,^11^, ^24^ and not so much a change from the first ICCR edition if the latter was already using AJCC TNM8 (depending on its timing). Spermatic cord invasion (invasion beyond the tunica vaginalis into the cord structures) defines pT3 disease and is a core element. This often means tumour present within or around the cord, or discontinuous tumour nodules in cord soft tissue. The dataset now provides a more precise definition: extension ‘beyond the angle of the epididymis and into the cord proper or tumour surrounding the vas deferens’ constitutes cord invasion (Figure 3). If there is tumour in lymphatics of the cord but not in the cord's stroma, that scenario is considered LVI (and categorised as pT2, not pT3) to avoid over‐staging. This nuance may be new in the 2nd edition, reflecting recent discussions outside the Dataset Advisory Committee (DAC) on how to stage ‘discontinuous’ tumour deposits in the cord vasculature.^25^, ^26^ Scrotal wall invasion (tumour extending through the tunica vaginalis into scrotal subcutis or skin) is pT4. The dataset continues to list it as a core item (because it changes stage to the highest T), although true scrotal invasion typically only occurs in neglected, large tumours or those with prior surgery (e.g., violation of scrotum facilitating spread). Regardless, the surgeon usually notes if the scrotum was involved; pathologists should comment on it if present (Figure 3).

- *Surgical margin status*: In a radical orchiectomy, the only pertinent surgical margin is the spermatic cord *proximal margin* (where the cord is transected). In a partial orchiectomy, there will be a parenchymal margin as well. The dataset retains margin status as a core element—one must state whether tumour is present at the (inked) cord margin or not, and for partial excisions whether the tumour is completely excised. A change in the second edition is more explicit guidance that LVI at the cord margin does not constitute a positive margin (it is still LVI but not an indication of tumour left in the patient, as long as the vessels were transected proximal to the tumour thrombus). Margin positivity is very rare in radical orchiectomies and there is little data on margin status as an independent prognostic factor apart from its correlation with pT category. Nonetheless, completeness of excision is a fundamental surgical pathology datum, so it remains core. The second edition did not significantly alter how margins are handled, but it provides these clarifications. For partial orchiectomy, the dataset emphasises careful evaluation of the tissue around the tumour to ensure no residual neoplasm is left in the remaining testis. If a margin is involved, that is critical to report (and would generally necessitate completion orchiectomy or further intervention).
- *Germ cell neoplasia in situ* (*GCNIS*): Although the presence of GCNIS in adjacent parenchyma does not directly affect staging or immediate prognosis, the dataset considers it a core item to report. This is because the absence of GCNIS in a tumour that otherwise looks like a typical GCT could signal an alternate diagnosis (e.g., a spermatocytic tumour or a sex cord tumour mimicking a GCT) or a rare non‐GCNIS‐derived GCT, which has different clinical implications. Essentially, reporting GCNIS confirms the origin of a classic GCT. If a specific form of germ cell neoplasm such as intratubular seminoma/embryonal carcinoma/yolk sac tumour is found, this should be reported. The second edition explicitly notes that GCNIS is the preferred term (replacing older terms like ‘intratubular germ cell neoplasia, unclassified’ and ‘carcinoma in situ of the testis’) to avoid confusion. In the context of an orchiectomy report, one should document if GCNIS is present (and it almost always will be for classic GCTs). If GCNIS is not found, the pathologist should be alerted to the possibility of a non‐GCNIS‐associated tumour type. This practice was likely encouraged in the first edition as well (since a 2015 consensus already highlighted it^1^), but the second edition places even greater emphasis on the need to mention GCNIS status. GCNIS itself is usually extensive in the testis harbouring a GCT (except in spermatocytic tumour, which lacks GCNIS, or prepubertal‐type GCTs). Its presence does not change management because the entire testis is removed, but it is relevant for understanding tumour pathogenesis and for considering contralateral testis biopsy in some situations (patients with one GCT have a risk of contralateral GCNIS). The dataset's inclusion of GCNIS as the core underlines the completeness of pathological assessment.
- *Coexistent pathology*: The dataset encourages noting other pertinent findings such as ‘burnt‐out’ tumours (regressed scars in the testis), features of testicular dysgenesis syndrome (which may correlate with tumour development) or other abnormalities in the testis parenchyma. These remain non‐core data at present but can be used for further therapy decisions. There is no significant change here between editions, aside from updated references regarding cryptorchidism and other risk factors for GCT.
- *Ancillary studies*: The orchiectomy dataset acknowledges that most diagnoses can be made on H&E morphology, but immunohistochemical stains are often useful for difficult cases (for example, distinguishing seminoma from embryonal carcinoma or confirming GCNIS). The ISUP consensus on IHC in testis tumours provides guidelines (e.g., OCT4, CD30, CD117, PLAP, SALL4 as germ cell markers, etc.).^27^ This remains a non‐core section—ancillary studies are included if needed. The second edition also mentions 12p (i(12p)) FISH testing as a useful adjunct in some scenarios, such as confirming a tumour is a GCNIS‐related GCT (postpubertal type) versus a prepubertal‐type tumour, since i(12p) is a molecular hallmark of GCTs related to GCNIS.^28^ These ancillary tests were likely mentioned in the first edition too; the update is minor, simply reflecting continued recommendations to use them where appropriate. They are not required universally, particularly because not all centres have access, but labs are encouraged to apply them for challenging cases to ensure accurate classification.

### Clinical Relevance and Impact on Management

A primary goal of the ICCR datasets is to ensure that pathology reports contain all information necessary for optimal patient management and accurate staging. The revisions in the second edition of the Orchiectomy dataset enhance this objective by aligning pathological evaluation with key clinical decision‐making milestones.

- In Stage I disease (no overt metastases on imaging), the decision between active surveillance versus adjuvant therapy (chemotherapy or radiotherapy for seminoma or chemotherapy/retroperitoneal lymph node dissection for NSGCT) heavily relies on pathological risk factors. A meticulously reported orchiectomy can stratify patients: for example, a seminoma patient with a 1.2 cm tumour, no LVI, and no rete testis invasion may be considered low risk and a candidate for surveillance, whereas one with a 5 cm seminoma with stromal rete testis invasion might be offered adjuvant therapy due to higher relapse risk.^17^, ^18^ The updated dataset provides the evidence context so that pathologists understand which features are truly high‐risk. The inclusion of comments about new findings (such as no prognostic significance of pagetoid rete testis involvement in seminomas, or the potential greater importance of pre‐orchiectomy serum tumour markers in seminoma prognosis^5^) helps prevent over‐calling risk. In NSGCT, features like LVI and embryonal carcinoma presence are highlighted because their presence could tilt the plan toward adjuvant chemotherapy rather than surveillance. The ICCR dataset thus functions as a checklist ensuring these critical factors are not missed. Indeed, studies show that when surveillance is employed in low‐risk NSGCT (no LVI, predominantly yolk sac or teratoma), outcomes are excellent, but if high‐risk features are present, about half will relapse if observed.^19^ Accurate pathology is the linchpin of this risk stratification.
- The ICCR Testis dataset is closely aligned with international clinical guidelines. The 2023 update of the European Association of Urology (EAU) guidelines on testicular cancer and the 2022 ESMO‐EURACAN clinical practice guideline both emphasise risk‐adapted management and the importance of pathology in decision‐making.^20^, ^21^ For instance, these guidelines acknowledge that in seminoma, tumour size >4 cm and rete testis invasion were historically used as risk factors, but emerging evidence (including from the large population studies cited in the dataset) suggests their prognostic value is modest; thus, surveillance can still be safe in their presence if imaging and markers are favourable. The ICCR notes echo this evidence‐based shift, ensuring pathologists do not overstate the risk of a seminoma solely due to rete testis invasion, for example. In NSGCT, guidelines essentially recommend adjuvant chemo if LVI is present because the relapse risk exceeds 50%—the dataset correspondingly treats LVI as a must‐report item.^4^ By dovetailing with these clinical consensus statements, the ICCR report format facilitates multidisciplinary discussions: everyone (urologists, oncologists, pathologists) has a unified understanding of the implications of the pathology report for the patient.
- Another area of clinical relevance is staging accuracy. The final composite stage of a testicular cancer patient (I, II, III) depends on pathological findings (pT and pN categories) combined with clinical factors (M category and S (serum tumour marker) category). The ICCR Orchiectomy dataset ensures that the pathologic components of staging are comprehensively evaluated.
- Ancillary prognostic information included in the dataset, even if non‐core, can support clinical management. For example, documenting the histology of uninvolved testis (amount of atrophy, presence of contralateral GCNIS or other abnormalities) can inform the need for endocrine follow‐up or fertility considerations. While that's not directly about cancer control, it speaks to the holistic management of the patient (e.g., patients with dysgenetic testes or certain syndromes might need contralateral biopsy or hormone monitoring). By encouraging a thorough examination (non‐core note), the dataset adds value beyond the cancer diagnosis itself.
- The 2nd edition Orchiectomy dataset underscores global applicability and resource considerations. They note that in some regions ancillary immunohistochemical or molecular tests may not be readily available; hence they list them as core only when absolutely necessary for diagnosis, otherwise as optional. For instance, i12p FISH is useful but not mandatory—the dataset includes it as a recommendation in Note 15 but not a core item.^29^ This ensures the dataset can be used in both high‐resource and limited‐resource settings without compromising the essential reporting standards. The consensus nature of ICCR (with authors from multiple countries) builds confidence that the recommendations are broadly acceptable and not just one institution's view.
- Research and quality improvement: By standardising what is reported, the dataset facilitates data collection for research and audits. It also identifies the reporting elements that need to be codified using SNOMED‐CT, thus making the data both computable and interoperable.^30^ If all pathology reports include, say, LVI status and stromal invasion of rete testis or pagetoid rete testis involvement consistently, large multicentre studies can be conducted or validated. The first edition ICCR dataset was used worldwide and helped in comparing outcomes; the second edition will continue to do so with the benefit of refined data points. This feedback loop (where practice standardisation enables further evidence generation) is explicitly part of ICCR's mission. Non‐core elements often highlight areas needing more evidence (e.g., is there prognostic significance to focal cord vascular invasion without cord tissue invasion?) The dataset provides what evidence exists^29^ and leaves it as a detail to monitor. Over time, today's non‐core might become tomorrow's core if evidence emerges—and the dataset architecture allows that evolution.

#### Remaining challenges and future directions

Despite the comprehensive nature of these datasets, some challenges remain in testicular tumour reporting:

- *Inter‐observer variability*: Even with a standard dataset, certain assessments can vary among pathologists. For example, deciding what constitutes ‘stromal invasion of rete testis’ vs. just pagetoid spread can sometimes be tricky, especially if tissue is distorted. The dataset definitions help, but training and experience are needed. Similarly, identifying LVI can be difficult; one pathologist might call something indeterminate while another calls it positive. The dataset tries to minimise equivocation by advising against reporting ‘suspicious LVI’—instead, if unsure, to do deeper levels or IHC or else call it not identified. This approach, if widely adopted, might reduce the ‘grey zone’ and yield clearer yes/no answers for LVI. Nonetheless, subtle LVI cases will remain diagnostically challenging. The potential use of ancillary IHC (endothelial markers) is mentioned but also cautioned that it's not routine practice everywhere.^23^
- *Rare variants and new entities*: The WHO 5th edition brought in some new entities (e.g., the rare prepubertal‐type neuroendocrine tumour of testis). The ICCR dataset lists them for completeness, but many pathologists may never see a case. If they do, this dataset ensures that they at least recognise it enough to name it. However, as new rare variants or molecular subtypes are discovered (for instance, the potential discovery of different molecular subgroups of seminoma or embryonal carcinoma), future editions will need to accommodate those. At present, histopathology remains the cornerstone, and the dataset is morphologically driven.
- *Communication and education*: Implementing the second edition will require dissemination and education in pathology communities. Pathologists who were used to the first edition or their own style must update their templates. Given that the differences are incremental (not a radical overhaul), this should be manageable. The ICCR and national societies will likely conduct webinars or issue summaries of changes. This manuscript itself serves an educational purpose, distilling the changes and their rationale for both pathologists and the broader oncology audience.
- *Monitoring outcomes and dataset effectiveness*: It would be beneficial to audit whether using the dataset improves patient outcomes. For example, does the use of ICCR synoptic reporting correlate with more appropriate use of adjuvant therapy in Stage I patients (reducing over‐ or under‐treatment)? Are there fewer instances of missing information in reports (which could otherwise lead to mismanagement)? These are areas where pathology as a field can collaborate with urologists and oncologists to measure impact. The references included (such as the TRISST trial on imaging^31^ or others on surveillance outcomes^32^, ^33^) remind us that pathology is one piece of the puzzle. The TRISST trial (which studied imaging frequency in seminoma surveillance) underscores that even for surveillance, having initial pathological risk factors documented is key to stratifying patients in trials. If pathology reports become more uniform, future clinical trials can better stratify or adjust for those factors, making results more reliable.
- *Future biomarker integration*: Markers like miRNA‐371 are on the horizon.^34^ If validated, they might reduce the reliance on some pathological risk factors—e.g., a low‐risk pathology but high miR‐371 post‐orchiectomy might suggest occult disease. Conversely, a high‐risk pathology but negative miR‐371 might justify surveillance. Future iterations of the ICCR dataset may incorporate molecular tests, or at minimum, make reference to them. This will obscure the boundaries between clinical and pathology data, requiring multidisciplinary consensus on how to incorporate new prognostic tools.

## Conclusion

In conclusion, the 2nd edition Orchiectomy dataset for testicular GCTs represents a significant step forward in standardising and refining pathology reporting for this disease. It embodies a formal, evidence‐backed consensus approach, ensuring that pathology reports deliver all the critical information needed for optimal patient management in a clear, structured format. The orchiectomy dataset's core data elements remain largely consistent with established practice: tumour type, size and extent (including specific invasions), LVI, margin status and GCNIS. From the perspective of an expert pathologist involved in their creation, this dataset will not only improve the quality of pathology practice but will also facilitate closer alignment between pathologists and treating clinicians. Ultimately, this should translate into better patient care—by informing treatment decisions (such as who can be safely observed versus who needs adjuvant therapy) and by contributing to consistent staging and prognostication. As medical knowledge advances—driven by updates to staging systems and WHO classifications—the ICCR will continue to evolve, with future updates anticipated. The current 2nd edition Orchiectomy dataset, however, offers a rigorously validated, internationally developed checklist that supports high‐quality, standardised reporting and promotes clinical confidence across diverse healthcare settings. Importantly, all ICCR datasets are freely available to the global pathology community via the ICCR website ([www.iccr‐cancer.org](http://www.iccr‐cancer.org)), reinforcing their role as a key international resource.

## Author contributions

FB and DMB wrote the initial draft manuscript with final review and revision by FW, GD, MTI, RJH, CSK, KM, MRR, ST, AY, JRS, TW and DMB. TW produced the figures.

## Conflict of interest

The authors do not report any conflicts of interest.

## Data Availability

The data that support the findings of this study are openly available in The International Collaboration on Cancer Reporting (ICCR) at https://www.iccr‐cancer.org/datasets/published‐datasets/urinary‐male‐genital/testis‐orchidectomy/.
